# PARP inhibitors promote stromal fibroblast activation by enhancing CCL5 autocrine signaling in ovarian cancer

**DOI:** 10.1038/s41698-021-00189-w

**Published:** 2021-06-09

**Authors:** Xiaoting Li, Tian Fang, Sen Xu, Ping Jin, Dongchen Zhou, Zhengzheng Wang, Huayi Li, Zongyuan Yang, Gang Chen, Xu Zheng, Yu Xia, Xiao Wei, Zeyu Zhang, Xin Yang, Ya Wang, Qinglei Gao

**Affiliations:** 1 Cancer Biology Research Center (Key Laboratory of the Ministry of Education), Tongji Hospital, Tongji Medical College, Huazhong University of Science and Technology, Wuhan, China; 2 Department of Hepatobiliary and Pancreatic Surgery, The Affiliated Tumor Hospital of Zhengzhou University, Henan Cancer Hospital, Zhengzhou, China

**Keywords:** Cancer therapeutic resistance, Targeted therapies, Ovarian cancer

## Abstract

Cancer-associated fibroblasts (CAFs) play significant roles in drug resistance through different ways. Antitumor therapies, including molecular targeted interventions, not only effect tumor cells but also modulate the phenotype and characteristics of CAFs, which can in turn blunt the therapeutic response. Little is known about how stromal fibroblasts respond to poly (ADP-ribose) polymerase inhibitors (PARPis) in ovarian cancer (OC) and subsequent effects on tumor cells. This is a study to evaluate how CAFs react to PARPis and their potential influence on PARPi resistance in OC. We discovered that OC stromal fibroblasts exhibited intrinsic resistance to PARPis and were further activated after the administration of PARPis. PARPi-challenged fibroblasts displayed a specific secretory profile characterized by increased secretion of CCL5, MIP-3α, MCP3, CCL11, and ENA-78. Mechanistically, increased secretion of CCL5 through activation of the NF-κB signaling pathway was required for PARPi-induced stromal fibroblast activation in an autocrine manner. Moreover, neutralizing CCL5 partly reversed PARPi-induced fibroblast activation and boosted the tumor inhibitory effect of PARPis in both BRCA1/2-mutant and BRCA1/2-wild type xenograft models. Our study revealed that PARPis could maintain and improve stromal fibroblast activation involving CCL5 autocrine upregulation. Targeting CCL5 might offer a new treatment modality in overcoming the reality of PARPi resistance in OC.

## Introduction

Ovarian cancer (OC) is the most devastating gynecologic malignancy, with the highest fatality rate^1^. Although traditional therapies including surgery and platinum-based chemotherapy have ameliorated OC prognosis, the occurrence of resistance to platinum, relapse, and metastasis still claim the lives of the majority of patients. The notorious prognosis of OC and the painfully slow progression of counteracting OC call for the development of novel therapeutic alternatives^2^.

OC is characterized by a high degree of heterogeneity that has long impeded optimizing the treatment of patients with this disease^2^. Recently, whole-genomic analyses have revealed that homologous recombination (HR) deficiency is a prevalent signature in high-grade serous OC^3^. Cancers with defective HR have been shown to be exquisitely sensitive to poly (ADP-ribose) polymerase inhibitors (PARPis) by inducing synthetic lethality^4,5^. This improved understanding of the molecular characterization of OC has contributed to incorporating PARPis into the management of OC^3,6,7^. It is estimated that >50% of OC patients likely benefit from PARPis^3–5^. Among PARPis, olaparib (Ola) was first approved by the US Food and Drug Administration to treat relapsed high-grade serous OC with germline BRCA1/2 mutations after progression to at least three prior lines of chemotherapy and has recently been approved for first-line maintenance treatment^8,9^. While PARPis have reshaped the landscape of OC management and clinical challenges, including acquired resistance in the overwhelming majority of patients, the paucity of biomarkers to define populations who can benefit from PARPis, and potential combination therapies to expand the applications of PARPis, constantly drive ongoing investigations^5,10^. Nevertheless, our knowledge regarding this targeted therapy is confined to tumor cells and remains insufficient in the tumor microenvironment (TME).

The TME, encompassing tumor cells, fibroblasts, immune cells, and extracellular matrix (ECM), has attracted much attention for its crucial role in tumor progression. There is increasing evidence revealing that conventional and targeted anticancer therapies could modulate the TME^11–13^. Traditional chemotherapy destroys subsets of cancer cells and simultaneously activates the stroma to promote cancer recurrence and metastasis^14,15^. In addition to eliminating HR-deficient tumors, PARPis could influence noncancerous cells such as by inducing genomic instability in normal cells^16^. PARPis have also been reported to participate in the development of tumor immunosuppression, resulting in preclinical combinatory use of PARPis and immunomodulators (e.g., programmed cell death protein-1/programmed cell death ligand-1 inhibitors) in breast cancer and OC^17,18^. Cancer-associated fibroblasts (CAFs) are the dominant resident cells in the TME and have versatile functions, including matrix deposition and remodeling, extensive reciprocal signaling interactions with cancer cells, and crosstalk with infiltrating leukocytes^19^. In confrontation with traditional chemotherapy, CAFs can be activated, which alters their features and enables them to fuel cancer progression^13–15,20^. The extrapolation that PARPis also engage in modulating the TME, especially CAFs, is reasonable, but supporting evidence is sparse.

In this study, we aimed to investigate how PARPis modulate stromal fibroblasts in OC. We observed that stromal fibroblasts were not susceptible to PARPis. Conversely, stromal fibroblasts were activated after long-term exposure to PARPis. In addition, we uncovered that PARPi-challenged fibroblasts displayed a unique secretory profile, where CCL5 was the most prominently upregulated. We also demonstrated that C-C chemokine motif ligand 5 (CCL5) is responsible for maintaining CAF activation, and neutralizing CCL5 could attenuate PARPi-induced CAF activation in vitro and in both BRCA1/2-wild type and BRCA1/2-mutant xenograft models and boost the tumor inhibitory effect of PARPis. Importantly, the nuclear factor (NF)-κB pathway is involved in regulating CCL5 secretion in PARPi-primed CAFs. Our results addressed the side effects of PARPis on the TME of OC, and effective blockade of these side effects could augment the efficacy of PARPis with the current therapeutic regimen.

## Results

### OC stromal fibroblasts are inherently not vulnerable to PARPis

**Fig. 1:**
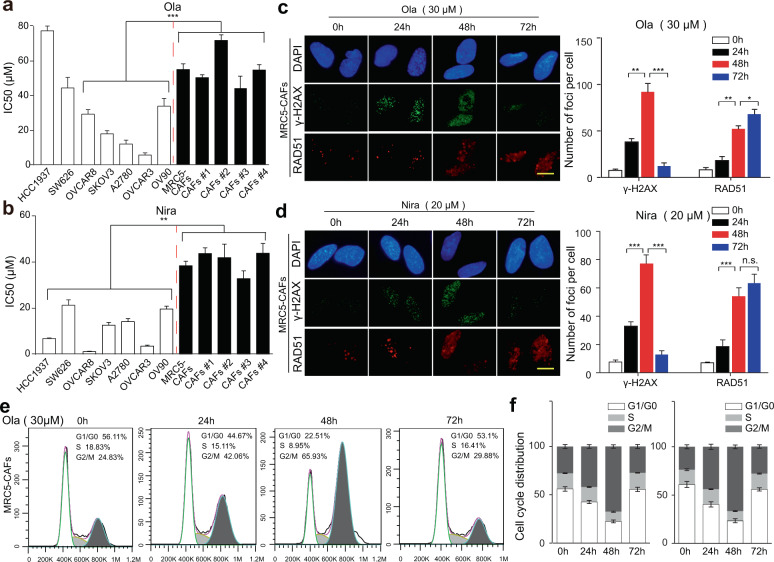
OC stromal fibroblasts are inherently not vulnerable to PARPis. **a**, **b** OC cell lines (SKOV3, OVCAR3, OVCAR8, A2780, OV90, and SW626), breast cancer cell line HCC1937, and CAFs (MRC5-CAFs and four primary CAFs) were treated with a range of concentrations of Ola or Nira for 72 h and were then assayed using CCK-8 for cell viability and IC50. **c**, **d** Representative microphotographs and the quantification of RAD51 and γ-H2AX foci formed in MRC5-CAFs upon exposure to 30 μM Ola or 20 μM Nira for the indicated periods. Scale bar, 5 µm. **e**, **f** Representative images and the quantification of changes in the cell cycle distribution of CAFs treated with 30 μM Ola for the indicated periods. Data are expressed as mean ± s.e.m., *p < 0.05; **p < 0.01; ***p < 0.001.

The biological rationale of PARPi application in OC relies on intrinsically high proliferative rates, genomic instability, and damaged DNA repair mechanisms in tumor cells^4^. In contrast to tumor cells, mutations are not commonly found in stromal fibroblasts^21^. To evaluate the sensitivity of cancer cells and stromal fibroblasts to PARPis, five human BRCA1/2-wild type OC cell lines, SKOV3, OVCAR3, A2780, OVCAR8, and OV90; one strain of BRCA1/2-mutant OC cell line, SW626; and one strain of BRCA1/2-mutant breast cancer cell line, HCC1937; MRC5-CAFs; and four other primary CAFs were exposed to various doses of PARPis. Representative immunofluorescence images identifying the CAFs by staining the traditional CAF marker alpha-smooth muscle actin (α-SMA) are shown in Supplementary Fig. 1a. Ola and niraparib (Nira) were chosen for their recognized effects in variant OC clinical trials^22,23^. The results showed that the IC50 values of PARPis in fibroblasts were generally higher than those in tumor cell lines, except for HCC1937 cells that did not respond to Ola as previously reported^24^ (Fig. 1a, b). Then we analyzed the expression patterns of HR-related signatures in the tumoral and stromal compartments of OC in the microdissected ovarian profile GSE 40595 (Supplementary Fig. 1b) and found that genes such as PTEN and ATM mediating PARPi resistance were highly expressed in the OC stroma. Next, dual immunofluorescence of the DNA damage marker γ-H2AX and the double-strand break repair marker RAD51 were dynamically evaluated in fibroblasts exposed to sublethal concentrations of Ola (30 μM) and Nira (20 μM), respectively (Fig. 1c, d). The foci of γ-H2AX were detected at the earliest time point of 24 h after treatment and continued to increase until 48 h. From 48 h, the number of γ-H2AX foci began to decline until they were not detectable at 72 h, in line with the dynamic alteration of G2/M cell cycle arrest (Fig. 1e, f and Supplementary Fig. 1c, d). However, for RAD51, we observed that its foci number increased with exposure time until a plateau period at 48 h but never dropped. Altogether, these data demonstrate that stromal fibroblasts are relatively insensitive to PARPis due to their competent HR function and timely escape of cell cycle arrest.

### PARPis promote the activation of OC stromal fibroblasts in vitro

**Fig. 2:**
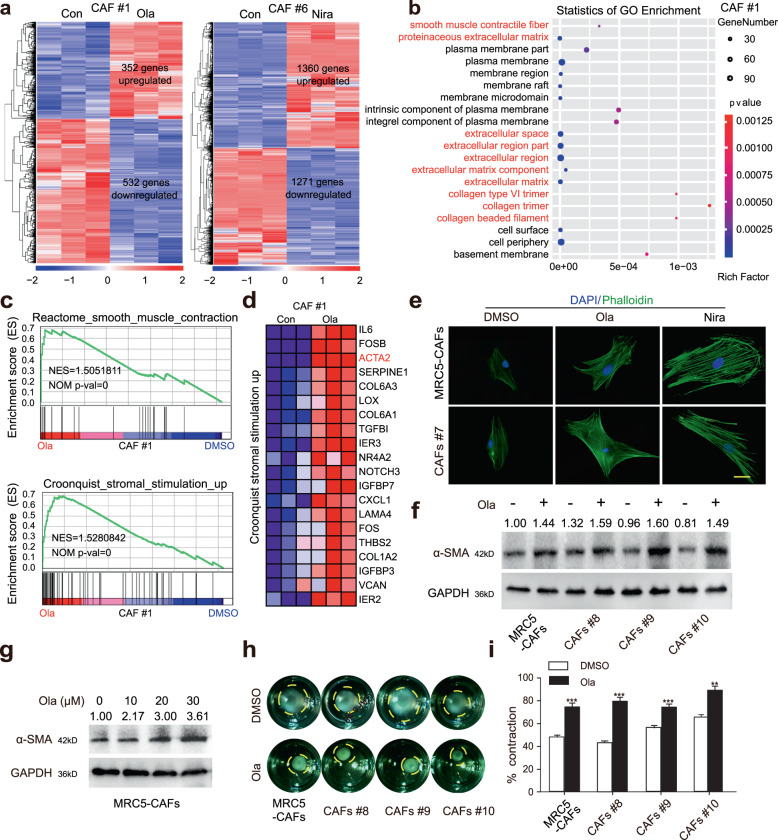
PARPis promote the activation of OC stromal fibroblasts in vitro. **a** Hierarchical clustering analysis results of RNA-seq in the PARPi-treated primary CAFs and control groups. **b**, **c** Gene ontology (GO) and GSEA showing significant enrichment for genes encoding ECM processes and signatures representing CAF activation in Ola-treated CAFs. **d** GSEA-derived heatmap showing the relative mRNA expression of the stroma activation signature genes. **e** Representative photographs of the cellular cytoskeleton by F-actin staining in MRC5-CAFs and primary CAFs after treatment with PARPis and their corresponding controls. The nuclei were counterstained with DAPI. Scale bar, 10 µm. **f**, **g** Immunoblotting of α-SMA in MRC5-CAFs or primary CAFs after 72 h of treatment with Ola. GAPDH served as the loading control. Relative densitometry values are labeled in the photographs. **h**, **i** Representative images and quantification of the collagen contraction capacity of the MRC5-CAFs and the primary CAFs in the control and Ola-treated groups. Data are expressed as mean ± s.e.m., *p < 0.05; **p < 0.01; ***p < 0.001.

The ability of stromal fibroblasts to adapt to stress engendered by cancer-targeted therapies may represent a key mechanism of resistance that, if effectively blocked, could lead to cancer cell death and improved patient outcomes^20,25,26^. To explore the adaptive responses of stromal fibroblasts under PARPi stress, we performed RNA-seq analysis in primary CAFs before and after treatment with PARPis. There were 884 and 2631 significantly altered genes in the Ola and Nira-treated groups, respectively (Fig. 2a). The gene ontology (GO) analysis revealed that the upregulated genes were mainly related to extracellular regions and ECM components (Fig. 2b and Supplementary Fig. 2a). Gene set enrichment analysis (GSEA) results showed that PARPis increased the stromal activation degree and the transcription of the most common CAF marker α-SMA (also known as ACTA2) (Fig. 2c, d and Supplementary Fig. 2b, c). Quantitative real time–PCR (qRT-PCR) verified the increased transcription of several stromal-associated genes, including α-SMA and COL6A1 (Supplementary Fig. 2d). Thus, we postulated that PARPis could promote stromal fibroblast activation. To further verify our postulation in vitro, using immunofluorescence, we discovered that PARPi-challenged fibroblasts presented a more stretched stellate morphology (Fig. 2e). Immunoblotting revealed that α-SMA expression was obviously improved following PARPi treatment (Fig. 2f, g and Supplementary Fig. 2e). Additionally, collagen contraction assays showed that fibroblasts treated with PARPis displayed a notably increased capacity to contract ECM (Fig. 2h, i and Supplementary Fig. 2f). The above findings suggested that PARPis exerted a pro-activation role in vitro in stromal fibroblasts.

### PARPis promote the activation of OC stromal fibroblasts in vivo

**Fig. 3:**
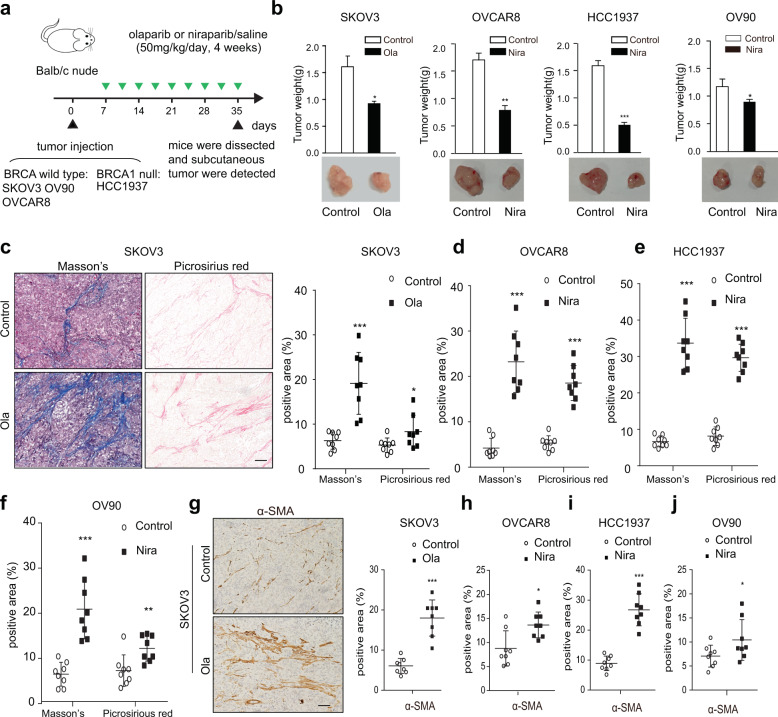
PARPis promote the activation of OC stromal fibroblasts in vivo. **a** The schematic diagram of subcutaneous tumor models evaluating the effect of Ola or Nira on OC stroma in vivo. **b** Representative images and weight quantification of subcutaneous tumors from mice (*n* = 8) bearing SKOV3, OVCAR8, HCC1937, and OV90 cells in the control and Ola or Nira-treated groups. **c**–**f** Representative photographs and quantification of Masson’s trichrome and picrosirius red staining showing the contents of stromal and collagen deposition in SKOV3, OVCAR8, HCC1937, and OV90 xenografts. Measures were taken from different samples (*n* = 8). Scale bar, 50 µm. **g**–**j** Representative IHC images and quantification of α-SMA in the Ola or Nira-challenged tumor stroma and the control stroma. Measurements were taken from distinct samples (*n* = 8). Scale bar, 50 µm. Data are expressed as mean ± s.e.m., *p < 0.05; **p < 0.01; ***p < 0.001.

To mimic and investigate the adaptive response of stromal fibroblasts caused by PARPis in vivo, we established three ovarian (SKOV3, OVCAR8, OV90) and one breast cancer (HCC1937) subcutaneous xenograft models in immunodeficient mice (Fig. 3a). Consistent with previous reports^27,28^ and the above Cell Counting Kit-8 (CCK-8) assays (Fig. 1a, b), PARPis showed evident role in inhibiting tumor growth while OV90 exhibited less sensitivity to Nira than other tumor cell lines (Fig. 3b). Interestingly, Masson’s trichrome and picrosirius red staining indicated that the tumors in the PARPi treatment group presented a more significant enrichment of stromal components and a robust increase in collagen abundance compared with those in the control groups (Fig. 3c–g). In addition, immunohistochemical (IHC) staining of α-SMA was more substantial in tumors exposed to PARPis (Fig. 3h–j). Although PARPi-induced killing effect on tumor cells might contribute to the relative increase of the stromal component, desmoplastic histological characteristics, such as increased interstitial spaces, excessive ECM deposition, and obvious derangement of stromal cells, suggested that PARPis exerted a pro-activation role in stromal fibroblasts in both BRCA1/2-wild type and BRCA1/2-mutant xenograft models.

### CCL5 secretion increases upon PARPi stimulation and correlates with the activation of OC stromal fibroblasts

**Fig. 4:**
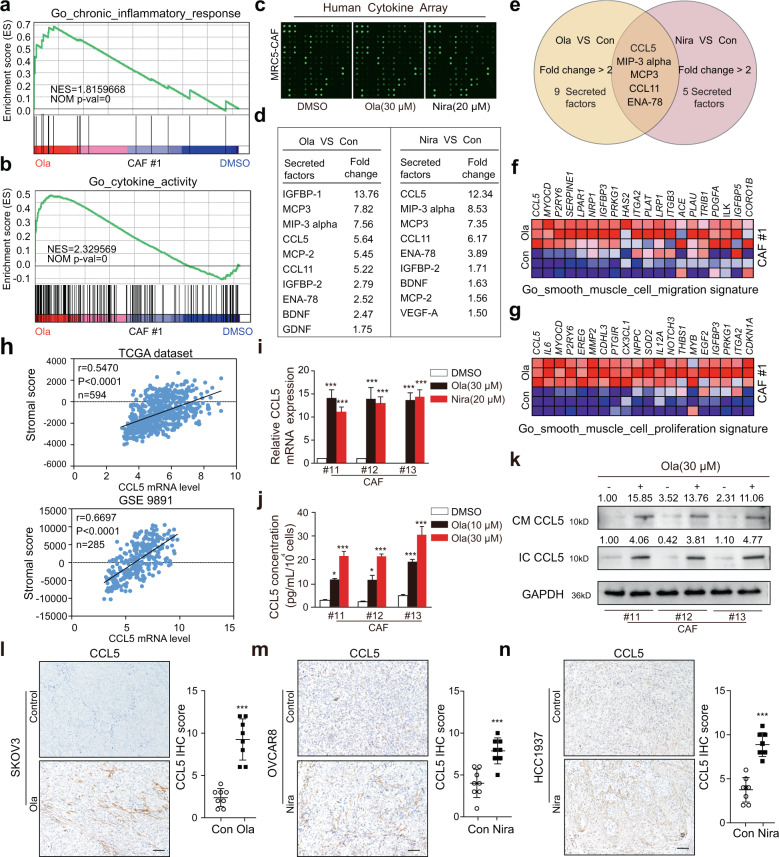
CCL5 secretion increases upon PARPi stimulation and correlates with stromal activation. **a**, **b** GSEA showing significant enrichment of the inflammatory response and cytokine activity signature in the Ola-treated CAFs. **c**, **d** Cytokine profiles from control MRC5-CAFs and MRC5-CAFs primed with either 30 μM Ola or 20 μM Nira for 72 h. The obviously elevated cytokines in the CM of MRC5-CAFs treated with Ola or Nira are listed. **e** Overlapping cytokines in the CM samples of MRC5-CAFs treated with Ola and Nira. **f**, **g** GSEA-derived heatmap showing the relative mRNA expression levels of smooth muscle cell activation-regulated genes in Ola-treated CAFs. **h** Spearman’s correlation analysis showing the relationship between CCL5 expression and the calculated stromal component score in the TCGA dataset, GSE 9891. **i** CCL5 mRNA expression by qRT-PCR in primary CAFs with or without PARPi treatment. **j** ELISA of CCL5 secreted by primary CAFs with or without PARPi exposure. **k** Immunoblotting of CCL5 protein in primary CAF extracellular conditioned medium (CM) or in cell lysates (IC) after PARPi exposure. GAPDH served as the loading control. Relative densitometric values are labeled in the photographs. **l**–**n** Representative IHC images and quantification of CCL5 protein expression in the Ola or Nira-challenged and control xenograft tumor stroma. Measurements were taken from distinct samples (*n* = 8). Scale bar, 50 µm. Data are expressed as mean ± s.e.m., *p < 0.05; **p < 0.01; ***p < 0.001.

Numerous studies have revealed that stromal fibroblasts exposed to cancer-targeted genotoxic agents resulted in significant upregulation of inflammatory factors or cytokines, which in turn promoted the proliferation, invasion, and resistance of cancer cells^13,20,26,29^. In our study, GSEA showed that PARPis also increased the degree of inflammatory response and the cytokine secretion activity of OC primary fibroblasts (Fig. 4a, b). To clarify these specific inflammatory cytokine secretion alterations, we detected conditioned medium (CM) from MRC5-CAFs before and after PARPi treatment using a human inflammatory cytokine array (Fig. 4c). A total of 10 cytokines were significantly increased (fold change >1.5) in CM from Ola-primed MRC5-CAFs, and 9 cytokines were increased in CM from Nira-primed MRC5-CAFs (Fig. 4d). Among these altered cytokines, five of the most upregulated cytokines, CCL5, MIP-3α, MCP3, CCL11, and ENA-78, were shared by both PARPis (Fig. 4e). The GSEA results of our RNA-seq data showed that CCL5 was elevated in the PARPi-treated group and was most relevant to smooth muscle cell migration and proliferation (Fig. 4f, g). The single-sample GSEA (ssGSEA) results of public data demonstrated that CCL5 mRNA expression was positively related to the stromal activation score as well as α-SMA, FAP, and FSP1 expression (Fig. 4h and Supplementary Fig. 3a, b). PARPi treatment increased intracellular CCL5 expression and CCL5 secretion in primary CAFs (Fig. 4i–k). However, negligible induction of CCL5 expression in cancer cells was observed (Supplementary Fig. 3c–f). Simultaneously, we confirmed that CCL5 was significantly upregulated in the tumoral stroma of both BRCA1/2-wild type and BRCA1/2-mutant xenografts after PARPi treatment (Fig. 4l–n and Supplementary Fig. 3h). It is worth mentioning that not only more positive area of CCL5 along with more stroma proportion but also increased intensity of CCL5 staining in the stromal area could be observed in PARPi-treated tumors (Supplementary Fig. 3g, h). Therefore, IHC score, synthetically estimating both the positive area and staining intensity, can be more appropriate than quantification of positive area. The above results indicated that CCL5 secretion increases upon PARPi stimulation and probably correlates with stromal activation.

### Autocrine CCL5 is required for PARPi-induced activation of OC stromal fibroblasts

**Fig. 5:**
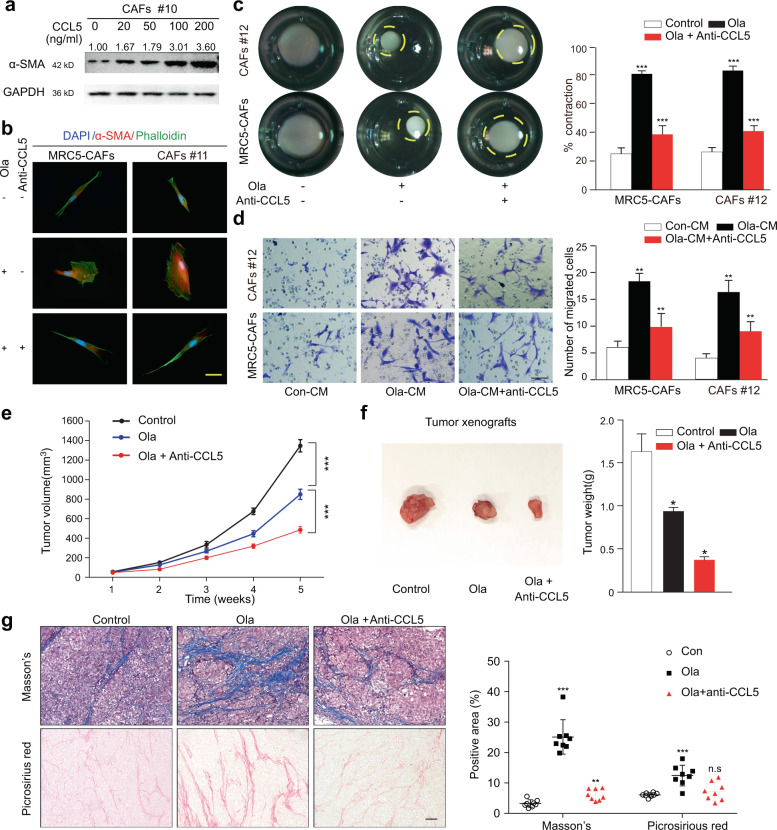
CCL5 is required for PARPi-induced stromal fibroblast activation. **a** Immunoblotting of α-SMA in primary CAFs after exogenous CCL5 administration. GAPDH served as the loading control. Relative densitometric values are labeled in the photographs. **b** The CCL5 neutralizing antibody suppressed the α-SMA elevation and cytoskeletal stretch of MRC5-CAFs and primary CAFs in response to Ola. Scale bar, 10 µm. **c** Representative images and quantification of the collagen contraction assays of MRC-CAFs and primary CAFs under treatment of Ola with or without CCL5 neutralizing antibodies. **d** Representative images and quantification of CAFs transwell assays using conditional medium derived from CAFs under treatment of Ola with or without CCL5 neutralizing antibodies. **e** Tumor volumes (mm^3^) of SKOV3 xenografts estimated using calipers per week for 35 days after tumor cell injections. (*n* = 8 mice per group). **f** Bright field images and weight quantification of subcutaneous tumors from mice (*n* = 8) injected intraperitoneally with vehicle or Ola or the combination of Ola and CCL5 neutralizing antibodies for 28 days after the tumor implantation. **g** Representative images and quantification of Masson’s trichrome staining and picrosirius red staining of the tumor tissue derived from SKOV3 xenografts under the indicated treatment. Measurements were taken from distinct samples (*n* = 8). Scale bar, 50 µm. Data are expressed as mean ± s.e.m., *p < 0.05; **p < 0.01; ***p < 0.001.

CCL5 has been previously reported to mediate microRNA-induced transformation of normal ovarian stromal fibroblasts into CAFs and was identified as an upstream regulator of inflammation^13,30^. To identify whether CCL5 was involved in PARPi-induced stromal fibroblast activation, we performed a series of experiments. First, immunoblotting revealed that exposure of stromal fibroblasts to recombinant CCL5 resulted in a continuous increase in α-SMA expression (Fig. 5a). The CCL5 neutralizing antibody significantly hampered the PARPi-induced activation of stromal fibroblasts (Fig. 5b, c and Supplementary Fig. 4a, b). In addition, CM from PARPi-treated fibroblasts recruited more stromal fibroblasts to the microenvironment, and the addition of CCL5 neutralizing antibody reduced this recruitment effect (Fig. 5d and Supplementary Fig. 4c). Most importantly, the addition of CCL5 neutralizing antibody significantly boosted the tumor-suppression role of PARPis in both BRCA1/2-wild type and BRCA1/2-mutant xenografts (Fig. 5e, f and Supplementary Fig. 4d, e), accompanied with partial reversal of PARPi-induced stromal activation (Fig. 5g and Supplementary Fig. 4f-h), indicating that this enhanced tumor growth inhibition may be partially due to the elimination of CCL5 exertion on sustaining stromal fibroblast activation. Altogether, these data suggested that PARPi-induced CCL5 expression and secretion were indeed involved in the maintenance of stromal fibroblast activation and neutralization of CCL5 boosted the antitumor efficacy of PARPis in both BRCA1/2-wild type and BRCA1/2-mutant xenograft models.

### PARPis induce CCL5 expression in CAFs through the NF-κB signaling pathway

**Fig. 6:**
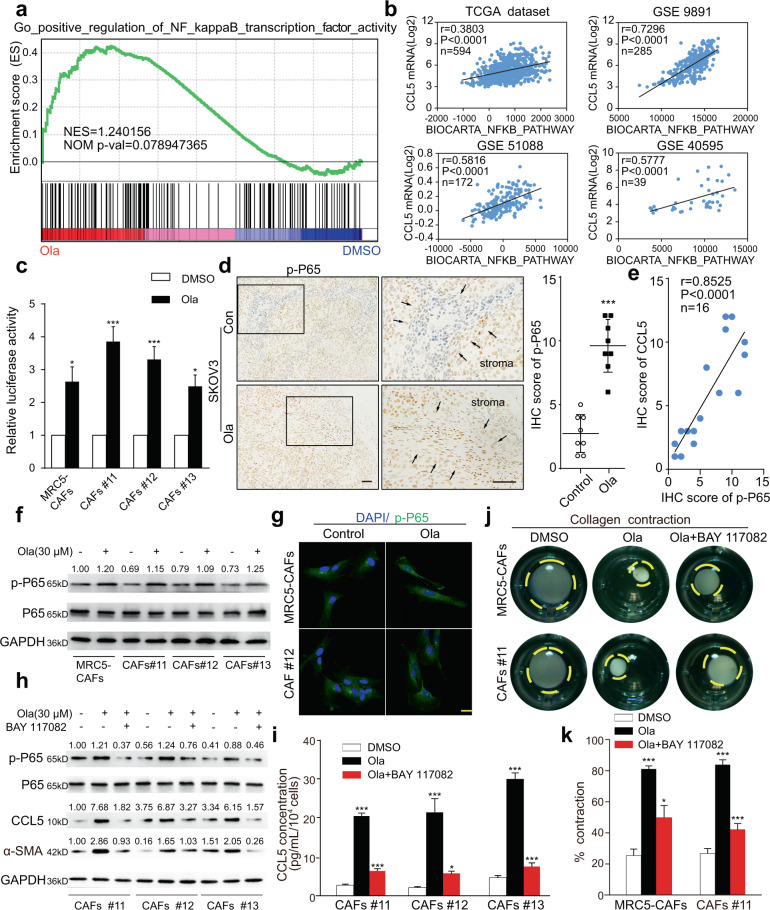
PARPis induce CCL5 expression in fibroblasts through NF-κB signaling. **a** GSEA of RNA-seq data showing significant enrichment for signature representing the NF-κB pathway activation in Ola-treated CAFs. **b** Spearman’s correlation analyses showing the relationship between calculated BIOCARTA_NFKB_PATHWAY score and CCL5 expression in the TCGA dataset, GSE 9891, GSE 51088, and GSE 40595. **c** The luciferase activity of p-NF-κB in CAFs with or without PARPi exposure. **d** Representative IHC images and quantification of p-P65 protein expression in the Ola-challenged and control tumor stroma of SKOV3 xenografts. Measurements were taken from distinct samples (*n* = 8). Scale bar, 50 µm. **e** The correlation analysis of the IHC scores of CCL5 and p-P65 among SKOV3 tumor xenografts. **f** Immunoblotting analysis of p-p65 in control and Ola-induced fibroblasts. GAPDH served as the loading control. Relative densitometric values are labeled in the photographs. **g** Representative immunofluorescence images of p-P65 in CAFs in the presence or absence of Ola. Scale bar, 10 µm. **h** Immunoblotting analysis of p-P65, CCL5, and α-SMA in control and Ola-challenged fibroblasts in the presence or absence of BAY 117082. GAPDH served as the loading control. Relative densitometric values are labeled in the photographs. **i** ELISA analysis of CCL5 expression in CM derived from CAFs under the indicated treatment. **j**, **k** Representative images and quantification of the collagen contraction assay of CAFs under the indicated treatment. Data are expressed as mean ± s.e.m., *p < 0.05; **p < 0.01; ***p < 0.001.

Based on a LASAGNA search and previous studies on the regulation of CCL5 transcription and expression^31–33^, NF-κB and activator protein 1 (AP-1) were found to be potential transcription factors for CCL5. Here GSEA results using our RNA-seq data showed that the NF-κB signaling pathway was significantly enriched in fibroblasts treated with Ola, while the AP-1 signaling pathway did not change significantly (Fig. 6a and Supplementary Fig. 5a). ssGSEA results showed that CCL5 mRNA levels were significantly correlated with NF-κB pathway activation scores in multiple OC databases (Fig. 6b). Therefore, we hypothesized that the NF-κB signaling pathway was involved in PARPi-induced CCL5 elevation in fibroblasts, and a series of experiments were performed to verify this hypothesis. First, a p-NF-κB reporter plasmid assay confirmed increased p-NF-κB pathway activity in stromal fibroblasts after PARPi treatment (Fig. 6c). IHC of xenograft tumor sections revealed that p-NF-κB protein was significantly elevated in the tumor stroma exposed to PARPis (Fig. 6d and Supplementary Fig. 5b, d). Additionally, correlation analysis of IHC scores demonstrated that CCL5 expression was markedly correlated with p-NF-κB expression in xenograft tumor stroma (Fig. 6e and Supplementary Fig. 5c). In vitro, PARPi treatment increased p-NF-κB protein expression in stromal fibroblasts (Fig. 6f and Supplementary Fig. 5e). Immunofluorescence experiments revealed that more p-NF-κB particles entered the nucleus after PARPi treatment (Fig. 6g). In addition, BAY-117082, a specific inhibitor of the NF-κB signaling pathway, effectively reduced the intracellular expression of p-NF-κB, CCL5, and α-SMA induced by PARPi (Fig. 6h and Supplementary Fig. 5f) and significantly reversed extracellular CCL5 upregulation in fibroblasts treated with PARPi (Fig. 6i and Supplementary Fig. 5g). Collagen contraction assays showed that PARPi-induced fibroblast-upregulated ability in contracting ECM was attenuated through inhibiting NF-κB pathway (Fig. 6j, k and Supplementary Fig. 5h). In conclusion, our results demonstrated that the NF-κB signaling pathway was involved in PARPi-induced CCL5 elevation in fibroblast activation.

### PARPis induce activation of stromal fibroblasts in human OC tumor specimens

**Fig. 7:**
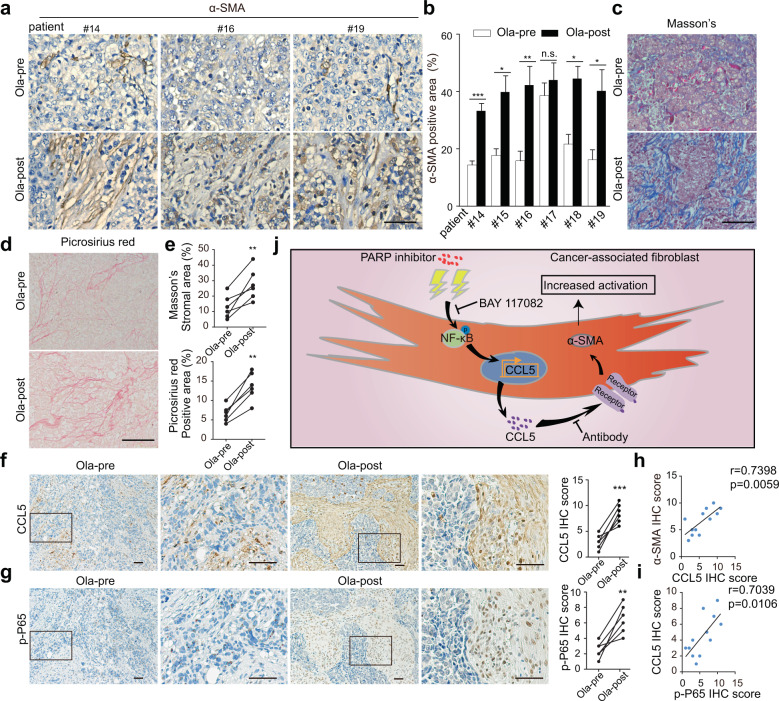
Olaparib induces stromal fibroblast activation in human OC tumor specimens. **a** Immunohistochemical detection of α-SMA in matched tumor specimens from patients with OC before and after administration with Ola. Scale bar, 50 µm. **b** Quantification of α-SMA-positive areas in tumor specimens from OC patients. Same images were measured repeatedly. **c**–**e** Representative images and quantification of Masson’s trichrome and picrosirius red staining showing that Ola administration increased the content of stromal components and collagen deposition in human OC tumor specimens. Scale bar, 50 µm. Same images were measured repeatedly. **f**, **g** Representative IHC images and quantification of CCL5 and p-P65 in matched tumor specimens from patients with OC before and after administration with Ola. Scale bar, 50 µm. Measures were taken from different samples (*n* = 6). **h** Correlation analysis of CCL5 IHC scores and α-SMA IHC scores in matched tumor specimens from patients with OC before and after administration with Ola. **i** Correlation analysis of p-P65 IHC scores and CCL5 IHC scores in matched tumor specimens from patients with OC before and after administration with Ola. **j** Schematic representation of the effect of PARPis on human ovarian stromal fibroblasts. Data are expressed as mean ± s.e.m., *p < 0.05; **p < 0.01; ***p < 0.001.

To extend these experimental observations to the clinical paradigm, we analyzed matched tumor specimens from six patients with OC before and after treatment with PARPis, and the corresponding clinical data are summarized in Supplementary Table 1. The Ola-post tumor specimens were collected during the second surgery after at least 6 months of Ola maintenance and Ola was stopped within 3 days before the surgery. Details about the treatment of patient#14 and the collection of corresponding tumor specimens are exhibited in the Gantt chart (Supplementary Fig. 6). The results of IHC showed that α-SMA was significantly increased in tumor specimens after PARPi administration (Fig. 7a, b). Masson’s trichrome and picrosirius red staining revealed that tumor specimens after PARPi administration had more abundant stroma accumulation (Fig. 7c–e). Further IHC analysis showed higher expression of CCL5 and p-NF-κB in tumor specimens after PARPi therapy (Fig. 7f, g). Furthermore, correlation analysis of IHC score showed that CCL5 expression was positively correlated with α-SMA expression, and p-NF-κB expression was positively correlated with CCL5 expression (Fig. 7h, i). Collectively, the above results suggested that PARPi promoted stromal fibroblast activation in human OC tumor specimens.

## Discussion

The majority of studies on PARPis have focused on defining appropriate candidates for them and expanding their applicability^5^. Herein we extended the investigation of PARPis from cancer cells to stromal fibroblasts and discovered that stromal fibroblasts were intrinsically invulnerable to PARPis. Intriguingly, PARPis were found to promote stromal fibroblast activation in vitro and in both BRCA1/2-wild type and BRCA1/2-mutant xenograft models, which was further validated in clinically matched specimens derived from patients who had undergone administration of Ola until disease progression. PARPi-primed fibroblasts exhibited a specific secretory profile, in which CCL5 was identified to be required for PARPi-induced fibroblast recruitment and activation. Furthermore, neutralization of CCL5 blunted PARPi-driven fibroblast activation and boosted the cancer suppression efficiency of PARPis in BRCA1/2-wild type and BRCA1/2-mutant OC xenograft models. Further results implied that the NF-κB pathway was involved in PARPi-induced secretion of CCL5 and that targeting this pathway could effectively reverse CCL5 secretion and decrease the continued activation of stromal fibroblasts (Fig. 7j). Together, our work uncovered a side effect of PARPis on tumor stromal fibroblast activation, suggesting that overcoming this “side effect” could potentially sensitize PARPis in OC.

As the most paradigm-shifting breakthrough in OC management, PARPis have made great strides in OC. Ola was the first PARPi approved for the treatment of advanced epithelial ovarian cancer (EOC) in patients harboring germline BRCA mutations and is now used for maintenance treatment of BRCA mutated advanced EOC patients^8,9^. Importantly, Nira was approved for maintenance treatment of patients with platinum-sensitive recurrent EOC who have achieved a complete or partial response regardless of BRCA status^23,34^. As the widespread use and great benefits of Ola and Nira have been witnessed, the impacts of Ola and Nira on stromal fibroblasts are worth exploring. Here we observed that PARPis abnormally increased stromal fibroblast activation in vitro and in both BRCA1/2-wild type and BRCA1/2-mutant xenograft models. More importantly, matched post-treatment tumor specimens showed significantly elevated α-SMA and more abundant stromal content than pre-treatment specimens, indicating that PARPi-induced stromal activation also occurred in patients receiving PARPis. Activated CAFs are characterized by a more stretched morphology, upregulation of activated CAF markers, and a specific secretory phenotype and play important roles in cancer progression^35^. Activated CAFs mainly stem from local resident normal fibroblasts educated by cancer cells, rather than directly from normal fibroblasts that have undergone somatic genetic mutation^36,37^. Cancer cell-derived cytokines such as transforming growth factor (TGF)-β1, platelet-derived growth factor, and fibroblast growth factor-2 were reported to be capable of transforming normal fibroblasts to CAFs^38–41^. In addition to cancer cells, other resident cells in the TME, such as macrophages, were also proven to activate adjacent fibroblasts^42,43^. Strikingly, traditional chemotherapy reportedly triggers remodeling of the TME, including fibroblast transformation, thus promoting cancer recurrence and metastasis^14,15,44^. These observations of fibroblast activation induced by traditional chemotherapy and targeted drugs such as PARPis in our study implied that stromal fibroblasts vigorously responded to DNA damage agents. As coevolution of CAFs and cancer cells during tumorigenesis is fundamental for cancer progression, disruption of the drug–cancer microenvironment interplay could foster optimized therapeutic efficiency of traditional chemotherapy and targeted drugs.

Molecular alternations and functional consequences associated with chemotherapy-primed CAFs have been profoundly investigated^45^. Myriad studies have revealed that exposing stromal fibroblasts to cancer-targeted genotoxic agents resulted in significant upregulation of inflammatory factors or cytokines including interleukin (IL)-1β, matrix metalloproteinase-3, IL-6, WNT16B, and IL-8^13,20,26,29^. Increases in proinflammatory factors enhance tumor growth and metastasis in pancreatic cancer, fuel the proliferation and invasion of prostate cancer cells, and promote cell survival and resistance to apoptosis in prostate cancer^29,46^. Mechanistically, the chemotherapy-induced persistent DNA damage response accounts for the upregulation of inflammatory factors or cytokines in fibroblasts^47^. Similarly, PARPis were also observed to induce DNA damage in ovarian CAFs. However, unlike those facing traditional chemotherapies, PARPi-challenged CAFs could be liberated from this type of DNA damage and survive, which may be associated with factors including cell type and the duration, intensity, and nature of DNA damage. PARPi-treated CAFs in this study exhibited a specific secretory profile primarily characterized by upregulation of CCL5, MIP-3α, MCP3, CCL11, and ENA-78, in which CCL5 elevation was most prominent and was required for PARPi-induced fibroblast activation. Recently, PARPis were found to enhance CCL5 secretion in ERCC1-deficient non-small cell lung cancer by triggering the pTBK1/IRF3/NF-κB signaling cascade^48^. Similarly, our further results demonstrated that the NF-κB pathway was involved in the PARPi-induced CCL5 secretion in CAFs. In summary, the effects of chemotherapy and targeted therapy on CAFs implicated several limitations of current treatment and indicated that targeting stromal fibroblast activation is also an avenue for novel therapies.

Overcoming the host response induced by chemotherapy could substantially improve therapeutic outcome and patient survival. For instance, altering the dosing schedule of systemic chemotherapy from a maximum-tolerated dose to a low-dose metronomic (LDM) regimen was proven to temper the stromal response and treatment resistance^44^. The HMG-CoA reductase inhibitor simvastatin was shown to alleviate senescence-associated secretory phenotype in fibroblasts and to avoid their cancer-promoting effect in breast cancer^49^. To optimize the anticancer efficacy of PARPis in OC, it might be pivotal to develop adjuvant therapies that curb stromal fibroblast activation. Among the PARPi-induced secretion profile of CAFs, we found that elevation of CCL5 was the most prominent and that PARPi-driven fibroblast activation was dependent on increased CCL5. In addition, neither OC cells nor OC cells treated with PARPis showed remarked CCL5 secretion compared with CAFs. Blocking CCL5 successfully alleviated PARPi-induced fibroblast activation and sensitized PARPis in OC and BRCA1/2-mutant breast cancer xenograft models, indicating that potential strategies to curb fibroblast activation, including neutralizing CCL5, facilitate augmenting the efficacy of PARPis in OC and BRCA1/2-mutant breast cancers. Our findings added to the knowledge of PARPi exertion in the context of the stromal compartment, which helped further complete our recognition of the effect of PARPis in OC.

In conclusion, this study depicted the responses of OC stromal fibroblasts to PARPi that was distinct but highly complementary to its established role of causing synthetic lethality in HR-deficient cancer cells. PARPi-elicited specific secretory profile was proved to be required for PARPi-induced fibroblast activation. Together, our work uncovered a side effect of PARPi on stromal fibroblast activation, suggesting that overcoming this negative consequence could potentially sensitize PARPi in OC.

## Methods

### Cells

Human OC cell lines, including OV90, OVCAR3, A2780, OVCAR8, SKOV3, and SW626, and human breast cancer cell line, HCC1937, were purchased from ATCC (Rockville, MD, USA) and cultured in MCDB 105 and Medium 199 (1:1), Dulbecco’s Modified Eagle Medium (DMEM), RPMI-1640, RPMI-1640, McCoy’5A, L-15, and RPMI-1640 media, respectively. MRC-5 human lung fibroblasts were obtained from the cell bank of the Chinese Academy of Sciences. Primary OC stromal fibroblasts were isolated and purified from fresh cancer tissues of OC patients as previously described^50^, and baseline information is summarized in Supplementary Table 2, all participants provided written informed consent at recruitment, and the institutional ethics review committee of Tongji Hospital approved all study procedures for human subjects. MRC-5 cells were transformed into activated phenotype MRC5-CAFs by TGF-β1 (50 ng/ml). All fibroblasts were cultured in DMEM/F-12 (1:1) medium (Gibco). All the above growth media were supplemented with 1% penicillin/streptomycin (Thermo Scientific) and 10% fetal bovine serum (Gibco). All cells were cultured in a humidified atmosphere incubator with 5% CO_2_ at 37 °C. Mycoplasma testing (Lonza) was performed regularly in our institution, and all cell lines were tested once after thawing or isolation and before other experiments.

### Drug sensitivity assay

Cell viability was examined using a CCK-8 (Dojindo Laboratories, Kumamoto, Japan). A total of 1 × 10^4^ cells were seeded into 96-well plates per well followed by treatment with serial concentrations of Ola or Nira (MCE, Shanghai, China) for 72 h. After incubation with CCK-8, the number of viable cells was measured at an absorbance of 450 nm using a microplate reader (Bio-Rad Laboratories). Each assay was performed in triplicate (*n* = 3).

### IHC, picrosirius red staining, and Masson’s trichrome staining

First, paraffin-embedded tissue sections were deparaffinized in xylene. After antigen retrieval, the slides were treated using 3% H_2_O_2_, blocked with 5% bovine serum, and incubated with primary antibodies against α-SMA (ab5694, Abcam, USA, used at 1/200 dilution), CCL5 (710001, Invitrogen, USA, used at 1/20 dilution), and phosphorylated (p)-NF-κB(Ser536) (ab28856, Abcam, USA, used at 1/100 dilution) overnight at 4 °C. The next day, the slides were incubated with horseradish peroxidase (HRP)-conjugated secondary antibody and then treated using the DAB Kit (BD Bioscience). Subsequently, slides were counterstained with hematoxylin. These results were also reviewed and scored independently by two investigators based on the staining intensity and the positively stained areas. Masson’s trichrome and picrosirius red (Servicebio, Wuhan, China) staining was carried out on paraffin-embedded sections of xenograft tumors and human OC tumor specimens, and the results were analyzed using ImageJ as described previously^51^.

### Western blot analysis

Cell extracts were prepared in radioimmunoprecipitation assay lysis buffer (Beyotime, Shanghai, China) supplemented with protease inhibitor mixture (Roche). A total of 40 µg of protein for each sample was separated on 8–12% sodium dodecyl sulfate–polyacrylamide gel electrophoresis gels and then transferred to nitrocellulose membrane. Membranes were incubated with the indicated primary antibodies at 4 °C overnight, followed by secondary antibodies for 1 h. Antibodies against α-SMA (ab5694, used at 1/400 dilution) and CCL5 (ab18984, used at 1/1000 dilution) were obtained from Abcam Biotechnology (Abcam, CA, USA). Antibodies against glyceraldehyde 3-phosphate dehydrogenase (GAPDH) were obtained from Proteintech (Manchester, United Kingdom). The p-NF-κB p65 antibody (#3033, used at 1/1000 dilution) and total NF-κB p65 antibody (#8242, used at 1/1000 dilution) were purchased from Cell Signaling Technology (Beverly, MA, USA). Finally, the membranes were treated with the corresponding HRP-linked secondary antibody (Abcam, USA) and were then detected with an enhanced ECL system (Pierce). All blots were derived from the same experiment and then they were processed in parallel; un-cropped images of all blots are shown in Supplementary Fig. 7.

### Immunofluorescence analysis

Cells were fixed on coverslips with 4% paraformaldehyde and then permeabilized in 0.1% Triton X-100. Next, the cells were incubated with 5% bovine serum albumin and then treated with primary antibodies, such as α-SMA (ab124964, Abcam, USA, used at 1/250 dilution), p-NF-κB p65 antibody (#3033, CST, USA, used at 1/1600 dilution), RAD51 (ab133534, Abcam, USA, used at 1/1000 dilution), and γ-H2AX (ab22551, Abcam, USA, used at 1/200 dilution) overnight at 4 °C. The next day, the cells were incubated with secondary donkey anti-rabbit antibody (Alexa Fluor 594 conjugated, Antgene, China, used at 1/200 dilution) or secondary donkey anti-mouse antibody (Alexa Fluor 488 conjugated, Antgene, China, used at 1/200 dilution). Then coverslips were treated with Phalloidin-iFluor 488 Reagent (ab176753) and DAPI (Servicebio, Wuhan, China). The fluorescence images were photographed with an Olympus BX53 microscope (Olympus, Tokyo, Japan). Each experiment was conducted in triplicate (*n* = 3).

### Collagen gel contraction assay

An in vitro floating collagen matrix contraction model was utilized to determine the contractile capacity of fibroblasts as described in our prior study^52^. The images of gels were analyzed with the ImageJ software. All results are presented as a percentage of the original well area, and all contraction assays were performed in triplicate (*n* = 3).

### Cell cycle assay

For cell cycle analysis, the cells were trypsinized and fixed with 70% cold ethanol overnight at −20 °C. The next day, the cells were centrifuged and then suspended in phosphate-buffered saline containing 20 mg/ml propidium iodide solution (Roche Diagnostics) and 50 mg/ml RNAse A (Qiagen). Finally, the cell cycle status was detected using flow cytometry (Beckman Coulter), and the results were analyzed by the FlowJo software. Each experiment was conducted in triplicate (*n* = 3).

### Transwell migration assay

First, 5 × 10^4^ MRC5-CAFs or primary CAFs were seeded into the upper chamber of the Transwell apparatus, and 600 μl of CM collected from Ola or Ola with anti-CCL5 (Human CCL5 antibody, AF-278-NA, R&D Systems, USA), or dimethyl sulfoxide (DMSO)-treated MRC5-CAFs or primary CAFs, was added into the lower chamber of the 24-well plate. The upper chambers were removed and fixed with methanol for 30 min and then stained with 0.1% crystal violet for 20 min after 24 h of culture. Finally, cells that did not migrate and remained stuck on the inner side of the chamber were wiped using a cotton bud, and the remaining migrated cells on the external side were observed and analyzed under microscopy. Each experiment was conducted in triplicate (*n* = 3).

### Luciferase reporter assay

For the luciferase reporter assay, cells were prepared in a six-well plate and transfected with the p-NF-κB reporter plasmid (D2206, Beyotime, Shanghai). After 24 h of transfection, cells were either left untreated or treated with PARPis for 72 h. Then the luciferase activities were measured by a Firefly Luciferase Reporter Gene Assay Kit in accordance with the manufacturer’s instructions. Each measurement was performed in triplicate (*n* = 3).

### RT-PCR and RNA-seq

Total RNA was isolated using TRIzol reagents (Invitrogen) in accordance with standard manufacturer’s protocols. For RT-PCR analysis, complementary DNA was synthesized using Moloney Murine Leukemia Virus reverse transcriptase (Takara, Japan). RT-PCR was performed in triplicate (*n* = 3) using the Bio-Rad CFX96 system with SYBR Green. The relative mRNA expression levels were calculated using the comparative Cq method (ΔΔ Cq method) on the basis of GAPDH as the reference gene. List of the primer sequences are included in Supplementary Table 3. For RNA-seq analysis, total RNA was sent to the sequencing company for sequencing using the Illumina NovaSeq6000 sequencing platform. According to the |log2Ratio| ≥ 1 and *p* value <0.05 difference standards, edgeR R-3.3.3 was used to screen for differential genes. GO analysis was conducted using the topGO software. KOBAS 2.0 software was used to test the statistical enrichment of differentially expressed genes in Kyoto Encyclopedia of Genes and Genomes pathways.

### Human antibody array

Human antibody arrays were performed in accordance with the manufacturer’s instructions. Briefly, CM samples from different groups were collected in centrifuge tubes, and the cells remaining on the dish were counted to normalize CM volumes for cell number. The CM samples were clarified by brief centrifugation, diluted with serum-free medium to a concentration equivalent to 1.5 × 10^6^ cells per 1.5 ml, and applied to the human cytokine antibody array AAH-CYT-G5 (RayBiotech, Norcross, GA, USA). Signals of proteins were determined with a GenePix 4200 A professional microarray scanner.

### Enzyme-linked immunosorbent assay (ELISA)

CM samples from PARPi-treated CAFs and control CAFs were collected as described above and stored at −80 °C. The concentration of CCL5 in each supernatant was measured using an ELISA kit (R&D Systems, Minneapolis, MN) according to the manufacturer’s instructions. Absorbance at 450 nm was measured by a microplate reader (Bio-Rad Laboratories). Each measurement was performed in triplicate (*n* = 3).

### Animal assays

All animal experiments were conducted in compliance with approval of the Committee on the Ethics of Animal Experiments in Hubei Province. BALB/c nude mice (weight 20–23 g, 4–6 weeks) were purchased and were manipulated in laminar flow cabinets under specific pathogen-free conditions and used as a xenograft model. Twenty-four mice were injected subcutaneously with each tumor cell line (including SKOV3, OVCAR8, OV90, and HCC1937) for 2 × 10^6^ cell per mouse. Seven days after inoculation, mice were randomly assigned to the PARPi single agent group (*n* = 8), the control group (*n* = 8), and the combination treatment group (*n* = 8). For PARPi single agent group, mice were injected intraperitoneally with Ola (50 mg/kg/day) or Nira (50 mg/kg/day). Mice in the control group were injected intraperitoneally with equal volume of saline mixed with 1% DMSO. To verify the effect of CCL5 blockade on stromal activation, we added the combination treatment group of mice that were administered with intraperitoneal Ola (50 mg/kg/day) or Nira (50 mg/kg/day) combining with CCL5 neutralizing antibodies (1 mg/kg, twice a week) (AF-478, R&D Systems, USA). Every 7 days after cell injection, the subcutaneous tumor volume was measured. On day 35 after cell injection, all mice were humanely sacrificed under anesthesia. Macroscopic lesions were recorded and tumor nodules were dissected, weighed, and paraffin embedded for subsequent detection.

### Statistical analysis

All results were analyzed using GraphPad Prism 6.0 and are expressed as mean ± s.e.m. based on at least three independent experiments. Statistical significance between two groups was calculated by Student’s unpaired two-tailed *t* test. Comparisons between multiple groups were determined by one-way analysis of variance followed by Tukey’s post hoc test. *p* < 0.05 was considered as statistically significant (*), *p* < 0.01 was considered highly significant (**), and *p* < 0.001 was considered extremely significant (***).

### Reporting summary

Further information on research design is available in the Nature Research Reporting Summary linked to this article.

## Supplementary information

Supplementary Information

Supplementary Data 1

Reporting Summary

## Data Availability

The RNA-sequencing data can be accessed in the Gene Expression Omnibus (GEO) database with the accession code GSE164088 (https://www.ncbi.nlm.nih.gov/geo/info/linking.html). The figures associated with these data are Figs. 2a–d, 4a, b, f, g, and 6a and Supplementary Figs. 2a–c and 5a.

## Code availability

Gene expression data, including GSE40595, GSE115635, GSE51088, and GSE9891 profiling data, were obtained online from Gene Expression Omnibus (http://www.ncbi.nlm.nih.gov/geo). Signals were interpreted, normalized and log2 scaled as previously described^26^. The Cancer Genome Atlas (TCGA) data were obtained from the TCGA data portal (https://portal.gdc.cancer.gov). Pearson’s correlation analysis was performed to analyze the association between the gene expression of CCL5 and FAP, FSP1, and α-SMA. LASAGNA-search 2.0 was used to analyze the transcription factors of CCL5 (https://biogrid-lasagna.engr.uconn.edu/lasagna_search). GSEA was conducted using GSEA 2.2.2 downloaded from the Broad Institute (http://www.gsea-msigdb.org/gsea/index.jsp). The mentioned signatures in this article were downloaded from the MSigDB. ssGSEA was conducted on GenePattern (https://cloud.genepattern.org/gp/pages/index.jsf) to generate stromal activation scores and signaling pathway activation scores in the GSE40595, GSE115635, GSE51088, GSE9891 and TCGA datasets as described previously^7^. Pearson’s correlation analysis was used for further exploration.
