# Supplementary material for: PARP inhibitors promote stromal fibroblast activation by enhancing CCL5 autocrine signaling in ovarian cancer
Source: NPJ Precis Oncol. 2021 Jun 9;5:49. doi: 10.1038/s41698-021-00189-w (PMC8190269; doi:10.1038/s41698-021-00189-w)
Supplement: Supplementary file 1 — Supplementary Information [file 41698_2021_189_MOESM1_ESM.pdf]

Supplementary Figure 1

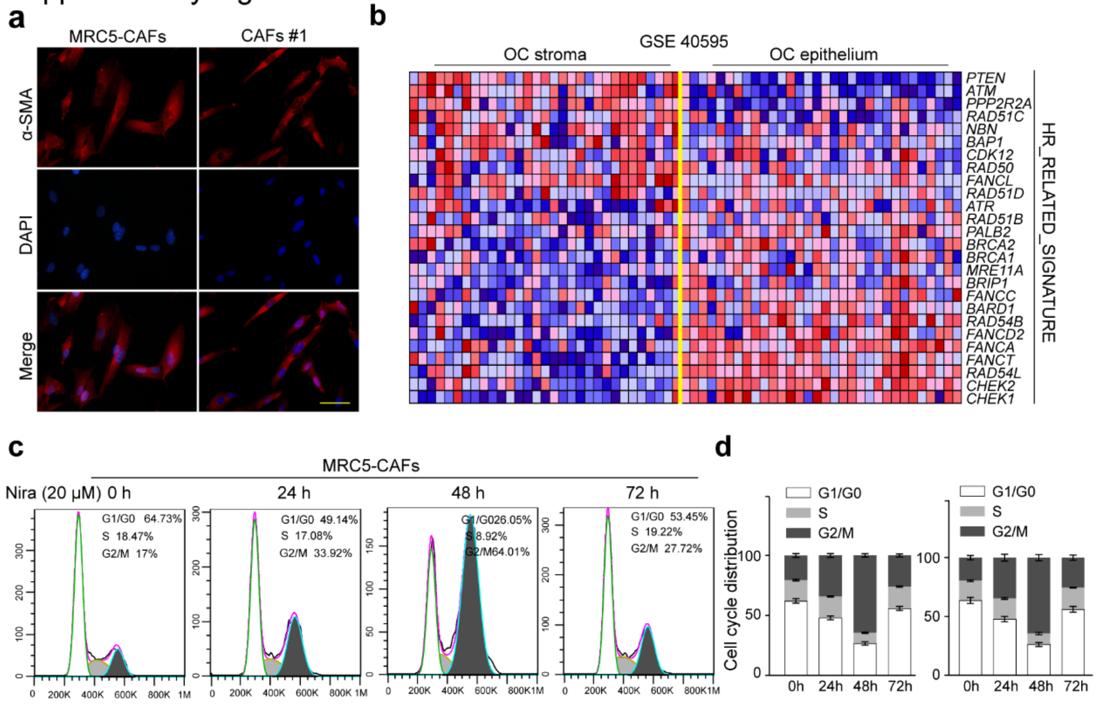


**Supplementary Figure 1.** PARPis induce a transient G2/M cell cycle arrest in CAFs. (a) Identification of MRC5-CAFs and primary CAFs isolated from patients without administration of chemotherapy by immunofluorescence staining of α-SMA. (b) GSEA-derived heatmap showing the relative mRNA expression differences of the HR-related signature between the OC stroma and the OC epithelium in GSE40595. (c-d) Representative images and the quantification of changes in the cell cycle distribution of CAFs treated with 20 μM Nira for the indicated periods.

Supplementary Figure 2

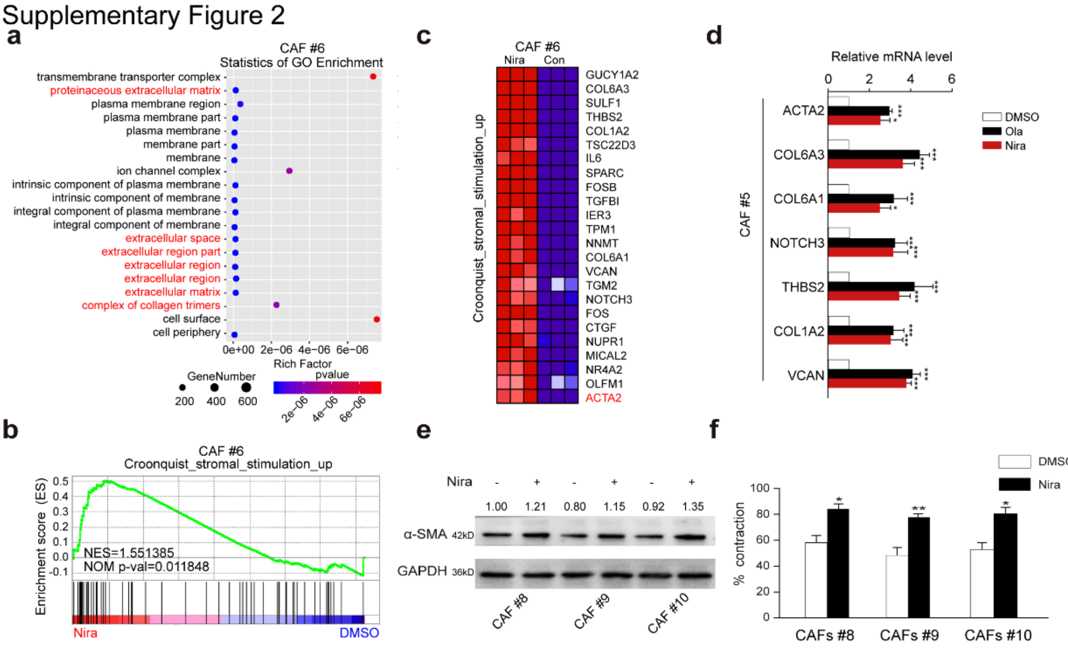

**Supplementary Figure 2.** Nira increases OC stromal fibroblast activation. (a) GO analysis showing significant enrichment of genes encoding ECM processes in Nira-treated CAFs. (b) GSEA showing significant enrichment of signatures representing CAF activation in Nira-treated CAFs. (c) GSEA-derived heatmap showing the relative mRNA expression of the stroma activation signature genes in Nira-treated CAFs. (d) qRT-PCR of representative upregulated genes in PARPi-treated primary CAFs. (e) Western blot analysis of α-SMA in primary CAFs after 72 h of treatment with 20 μM Nira. GAPDH served as the loading control. Relative densitometry are labeled in the photographs. (f) Quantification of the collagen contraction capacity of primary CAFs in the control and Nira-treated groups.

Supplementary Figure 3

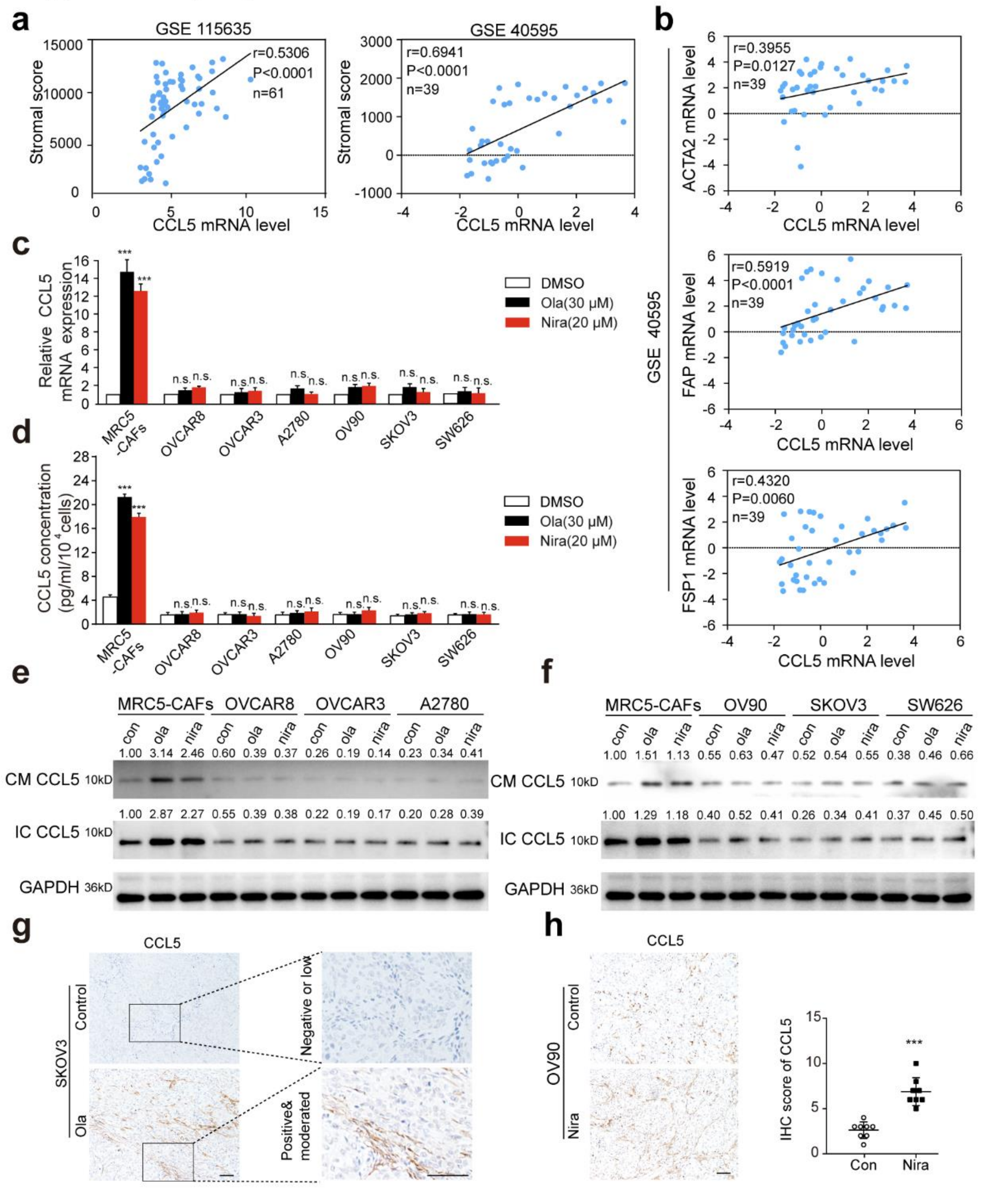


**Supplementary Figure 3.** CCL5 expression in the tumor stoma is associated with stromal activation in OC. (a) Spearman's correlation analysis showing the relationship between CCL5 expression and the calculated stromal component score in GSE115635 and GSE 40595. (b) Spearman's correlation analysis of CCL5 expression and classical fibroblasts activation markers as α-SMA, FAP and FSP1 in microdissected OC stroma profile GSE 40595. (c) qRT-PCR analysis of the relative gene expression of CCL5 in MRC5-CAFs and six OC cell lines, in the presence or

absence of PARPi. (d) ELISA of CCL5 secreted by MRC5-CAFs and six OC cell line with or without PARPi exposure. (e-f) Immunoblotting of CCL5 protein in extracellular conditioned medium (CM) or in cell lysates (IC) of MRC5-CAFs and six OC cell lines after PARPi exposures. (g) Representative photographs showing the positive/moderate and negative/low CCL5 expression in tumoral stroma of SKOV3 xenografts. Scale bar, 50 μm. (h) Representative IHC images and quantification of CCL5 protein expression in the Nira-challenged and control xenograft models bearing OV90. Measurements were taken from distinct samples (n=8). Scale bar, 50 μm. Data are expressed as the mean ± s.e.m, $*p < 0.05$; $**p < 0.01$; $***p < 0.001$.

Supplementary Figure 4

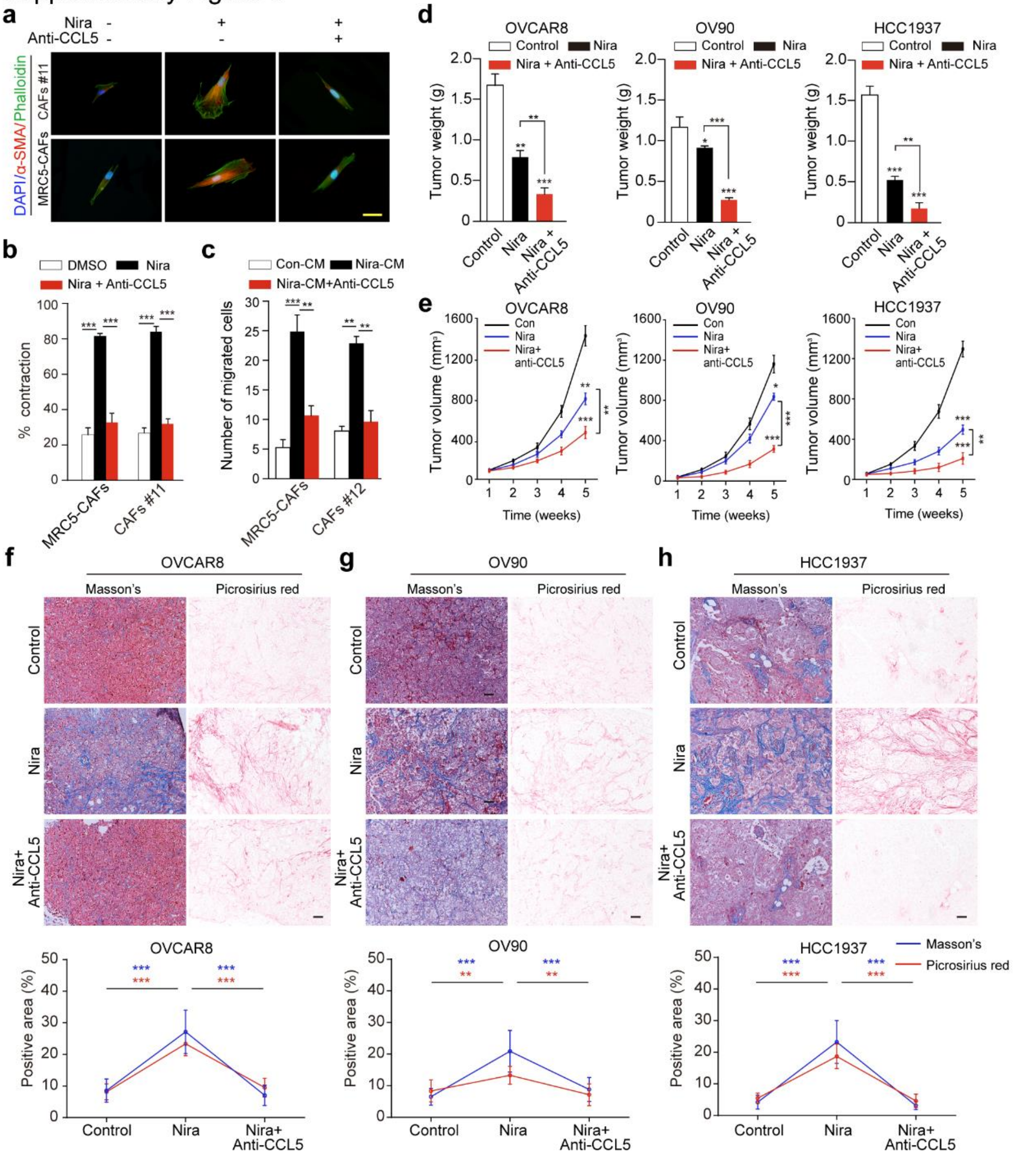


**Supplementary Figure 4.** CCL5 is required for PARPi-induced activation of CAFs in BRCA1/2-wildtype and BRCA1/2-mutant models. (a) The CCL5 neutralizing antibody suppressed the α-SMA increase and cytoskeletal stretch of MRC5-CAFs and primary CAFs in response to Nira. (b) Quantification of the collagen contraction assays of CAFs under the indicated treatment. (c) Quantification of the fibroblast migration experiments using CM derived from CAFs under the indicated treatment. (d) Weight quantification of xenograft models bearing OVCAR8, OV90, and HCC1937 tumors from mice (n=8) injected intraperitoneally with vehicle or Nia or the combination

of Nira and CCL5 neutralizing antibody for 28 days after tumor implantation. (e) Tumor volumes (mm3) estimated using calipers per week for 35 days after tumor cell injections. (n=8 mice/group) (f-h) Representative images and quantification of Masson’s trichrome and picrosirius red staining of tumor tissue derived from the mice models under the indicated treatment. Measures were taken from distinct samples (n=8). Scale bar, 50 µm. Data are expressed as mean ± s.e.m, *p < 0.05; **p < 0.01; ***p < 0.001.

Supplementary Figure 5

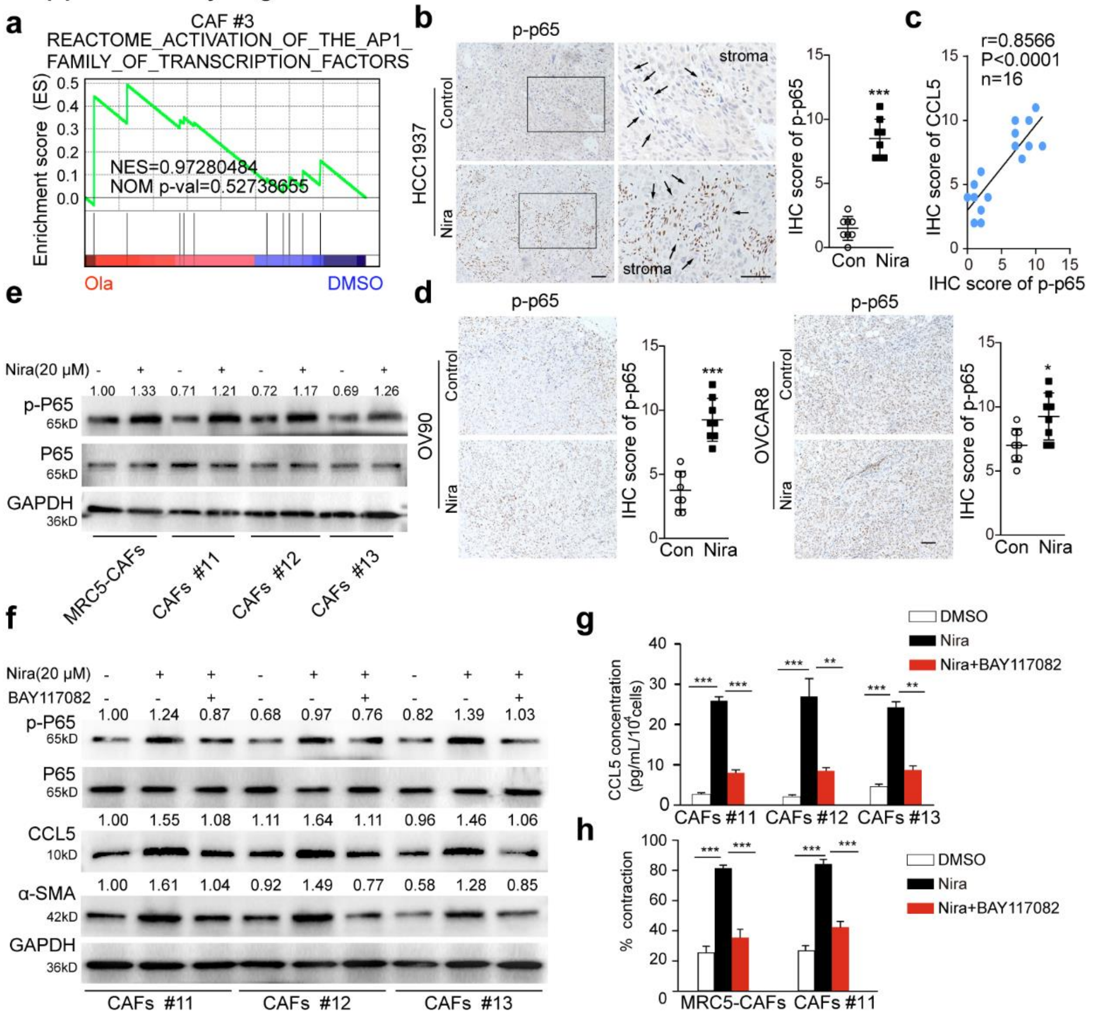


**Supplementary Figure 5.** Nira induces CCL5 expression in OC stromal fibroblasts through NF-κB signaling. (a) Results of GSEA analysis for the signature

"REACTOME_ACTIVATION_OF_THE_AP1_FAMILY_OF_TRANSCRIPTION_FACTORS" in RNA-seq data of CAFs treated with or without Ola. (b) Representative IHC images and quantification of p-P65 protein expression in the Nira-challenged and control tumor stroma of HCC1937 xenografts. Measurements were taken from distinct samples (n=8). Scale bar, 50 µm. (c) The correlation analysis of the IHC scores of CCL5 and p-P65 among HCC1937 tumor xenografts. (d) Representative IHC images and quantification of p-P65 protein expression in the PARPi-challenged and control tumor stroma of OV90 and OVCAR8 xenografts. Measurements were taken from distinct samples (n=8). Scale bar, 50 µm. (e) Immunoblotting analysis of p-P65 in control and Nira-challenged stromal fibroblasts. GAPDH served as the loading control. Relative densitometry values are labeled in the photographs. (f) Immunoblotting analysis of p-P65, CCL5, α-SMA in control and Nira-challenged fibroblasts, in the presence or absence of BAY 117082. GAPDH served as the loading control. Relative densitometry values are labeled in the photographs. (g) CCL5 protein level in CM derived from control and Nira-induced fibroblasts in the presence or absence of BAY 117082. (h) Quantification of the collagen contraction assay of CAFs under the indicated treatment. Data are expressed as mean ± s.e.m, *p < 0.05; **p < 0.01; ***p < 0.001.

Supplementary Figure 6

a

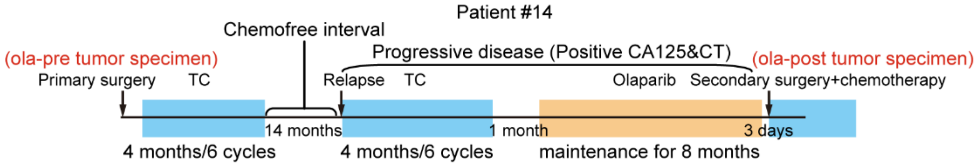


**Supplementary Figure 6.** (a) Gantt chart showing the treatment of patient#14 and details about the collection of Ola-pre and Ola-post tumor specimen.

Supplementary Figure 7： Un-cropped images

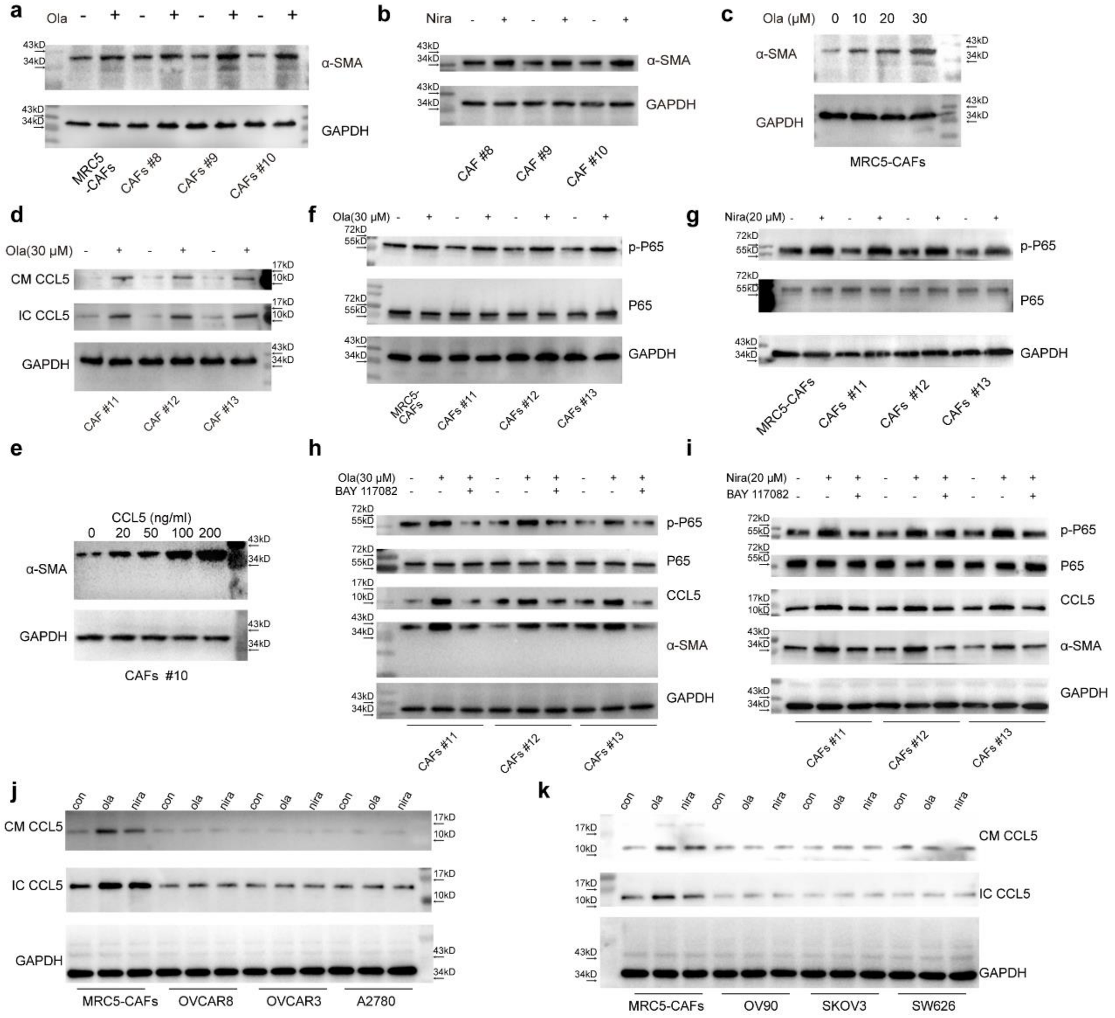


**Supplementary Figure 7： Uncropped images.** (a-k) Un-cropped images of the blots in Figure. 2f, Supplementary Figure 2e, Figure 2g, Figure 4k, Figure 5a, Figure 6f, Supplementary Figure 5e, Figure 6h, Supplementary Figure 5f, Supplementary Figure 3e, and Supplementary Figure 3f.

| SAMPLE | Age | Pathology | Primary Treatment (ola-pre tumor specimen) | Chemofree interval | Second line chemotherapy | Olaparib maintenace | Ola-free interval | Secondary surgery (ola-post tumor specimen) |
|---|---|---|---|---|---|---|---|---|
| **#14** | 43 | High grade papillary serous carcinoma | surgery+platinum-based chemotherapy | 14 months | 6 cycles | 8 months | 3 days | Yes |
| **#15** | 47 | High grade serous carcinoma | surgery+platinum-based chemotherapy | 16 months | 6 cycles | 9 months | 2 days | Yes |
| **#16** | 39 | Invasive high grade serous carcinoma | surgery+platinum-based chemotherapy | 28 months | 4 cycles | 18 months | 3 days | Yes |
| **#17** | 42 | High grade serous carcinoma | surgery+platinum-based chemotherapy | 20 months | 6 cycles | 15 months | 3 days | Yes |
| **#18** | 50 | Invasive high grade serous carcinoma | surgery+platinum-based chemotherapy | 7 months | 6 cycles | 6 months | 2 days | Yes |
| **#19** | 46 | High grade serous carcinoma | surgery+platinum-based chemotherapy | 12 months | 4 cycles | 12 months | 3 days | Yes |

**Supplementary Table 1:** Summary of matched OC tumor specimen information before and after Olaparib administration.

| SAMPLE | Age | Pathology | Tumor | Chemotherapy | Primary CAFs |
|---|---|---|---|---|---|
| **Patient 1** | 48 | High grade papillary serous carcinoma | Primary tumor | No | CAFs #1 |
| **Patient 2** | 67 | High grade serous carcinoma | Primary tumor | No | CAFs #2 |
| **Patient 3** | 59 | Invasive high grade serous carcinoma | Primary tumor | No | CAFs #3 |
| **Patient 4** | 43 | High grade serous carcinoma | Primary tumor | No | CAFs #4 |
| **Patient 5** | 53 | Invasive high grade serous carcinoma | Primary tumor | No | CAFs #5 |
| **Patient 6** | 54 | High grade serous carcinoma | Primary tumor | No | CAFs #6 |
| **Patient 7** | 48 | High grade serous carcinoma | Primary tumor | No | CAFs #7 |
| **Patient 8** | 50 | High grade serous carcinoma | Primary tumor | No | CAFs #8 |
| **Patient 9** | 57 | High grade papillary serous carcinoma | Primary tumor | No | CAFs #9 |
| **Patient 10** | 56 | High grade serous carcinoma | Primary tumor | No | CAFs #10 |
| **Patient 11** | 67 | Invasive high grade serous carcinoma | Primary tumor | No | CAFs #11 |
| **Patient 12** | 47 | High grade serous carcinoma | Primary tumor | No | CAFs #12 |
| **Patient 13** | 61 | High grade papillary serous carcinoma | Primary tumor | No | CAFs #13 |

**Supplementary Table 2:** Summary of primary CAF source information.

| Gene | Forward Sequence | Reverse Sequence |
|---|---|---|
| Human CCL5 | CCTGCTGCTTTGCCTACATTGC | ACACACTTGGCGGTTCTTTCGG |
| human ACTA2 | CTATGCCTCTGGACGCACAACT | CAGATCCAGACGCATGATGGCA |
| human COL6A3 | CCTGGTGTAACTGATGCTGCCA | AAGATGGCGTCCACCTTGGACT |
| human COL6A1 | GCCTTCCTGAAGAATGTCACCG | TCCAGCAGGATGGTGATGTCAG |
| Human NOTCH3 | TACTGGTAGCCACTGTGAGCAG | CAGTTATCACCATTGTAGCCAGG |
| human THBS2 | CAGTCTGAGCAAGTGTGACACC | TTGCAGAGACGGATGCGTGTGA |
| human COL1A1 | GATTCCCTGGACCTAAAGGTGC | AGCCTCTCCATCTTTGCCAGCA |
| human VCAN | TTGGACCTCAGGCGCTTTCTAC | GGATGACCAATTACACTCAAATCAC |

**Supplementary Table 3:** Listing of the primer sequences (5′–3′).
