# Supplementary material for: PARP inhibitors promote stromal fibroblast activation by enhancing CCL5 autocrine signaling in ovarian cancer
Source: NPJ Precis Oncol. 2021 Jun 9;5:49. doi: 10.1038/s41698-021-00189-w (PMC8190269; doi:10.1038/s41698-021-00189-w)
Supplement: Supplementary file 3 — Reporting Summary [file 41698_2021_189_MOESM3_ESM.pdf]

nature research

Corresponding author(s): Qinglei Gao

Last updated by author(s): Apr 8, 2021

Reporting Summary

Nature Research wishes to improve the reproducibility of the work that we publish. This form provides structure for consistency and transparency in reporting. For further information on Nature Research policies, see our Editorial Policies and the Editorial Policy Checklist.

Statistics

For all statistical analyses, confirm that the following items are present in the figure legend, table legend, main text, or Methods section.

n/a	Confirmed	
☐	☒	The exact sample size (*n*) for each experimental group/condition, given as a discrete number and unit of measurement
☐	☒	A statement on whether measurements were taken from distinct samples or whether the same sample was measured repeatedly
☐	☒	The statistical test(s) used AND whether they are one- or two-sided<br>*Only common tests should be described solely by name; describe more complex techniques in the Methods section.*
☒	☐	A description of all covariates tested
☒	☐	A description of any assumptions or corrections, such as tests of normality and adjustment for multiple comparisons
☒	☐	A full description of the statistical parameters including central tendency (e.g. means) or other basic estimates (e.g. regression coefficient) AND variation (e.g. standard deviation) or associated estimates of uncertainty (e.g. confidence intervals)
☒	☐	For null hypothesis testing, the test statistic (e.g. *F*, *t*, *r*) with confidence intervals, effect sizes, degrees of freedom and *P* value noted<br>*Give P values as exact values whenever suitable.*
☒	☐	For Bayesian analysis, information on the choice of priors and Markov chain Monte Carlo settings
☒	☐	For hierarchical and complex designs, identification of the appropriate level for tests and full reporting of outcomes
☒	☐	Estimates of effect sizes (e.g. Cohen's *d*, Pearson's *r*), indicating how they were calculated

Our web collection on statistics for biologists contains articles on many of the points above.

Software and code

Policy information about availability of computer code

Data collection	Gene expression data, including GSE40595, GSE115635, GSE51088, and GSE9891 profiling data, were obtained online from Gene Expression Omnibus (http://www.ncbi.nlm.nih.gov/geo). The Cancer Genome Atlas(TCGA) data were obtained from the TCGA data portal (https://portal.gdc.cancer.gov) using the published dataset. The mentioned signatures in this article were downloaded from the MSigDB(http://software.broadinstitute.org/gsea/msigdb/index.jsp) or published literatures.
Data analysis	GSEA was conducted using GSEA 2.2.2 from the Broad Institute (http://www.gsea-msigdb.org/gsea/index.jsp). ssGSEA was conducted on GenePattern (https://cloud.genepattern.org/gp/pages/index.jsf) to generate stromal activation scores and signaling pathway activation scores in the GSE40595, GSE115635, GSE51088, GSE9891 and TCGA datasets. Pearson's correlation analysis was performed to analyze the association between gene expression of CCL5 and FAP, FSP1, α-SMA. LASAGNA-search 2.0 was used to analyze the transcription factors of CCL5 (https://biogrid-lasagna.engr.uconn.edu/lasagna_search).

For manuscripts utilizing custom algorithms or software that are central to the research but not yet described in published literature, software must be made available to editors and reviewers. We strongly encourage code deposition in a community repository (e.g. GitHub). See the Nature Research guidelines for submitting code & software for further information.

Data

Policy information about availability of data

All manuscripts must include a data availability statement. This statement should provide the following information, where applicable:

- Accession codes, unique identifiers, or web links for publicly available datasets
- A list of figures that have associated raw data
- A description of any restrictions on data availability

The RNA-sequencing data can be accessed in the Gene Expression Omnibus (GEO) database with the accession code GSE164088 (https://www.ncbi.nlm.nih.gov/

geo/info/linking.html.). The figures associated with these data are Fig. 2a-d, Fig. 4a, b, f, g, Fig. 6a, Supplementary Fig. 2a-c, and Supplementary Fig. 5a.

Field-specific reporting

Please select the one below that is the best fit for your research. If you are not sure, read the appropriate sections before making your selection.

☒ Life sciences ☐ Behavioural & social sciences ☐ Ecological, evolutionary & environmental sciences

For a reference copy of the document with all sections, see nature.com/documents/nr-reporting-summary-flat.pdf

Life sciences study design

All studies must disclose on these points even when the disclosure is negative.

Sample size	Sample sizes were determined based on similar animal studies in previous work.
Data exclusions	No data were excluded from the analyses.
Replication	Experiments were replicated where applicable.
Randomization	Mice were randomly assigned to different groups before treatment.
Blinding	Investigators were blinded to the group allocation during data collection and analysis.

Reporting for specific materials, systems and methods

We require information from authors about some types of materials, experimental systems and methods used in many studies. Here, indicate whether each material, system or method listed is relevant to your study. If you are not sure if a list item applies to your research, read the appropriate section before selecting a response.

Materials & experimental systems

n/a	Involved in the study	
☐	☒	Antibodies
☐	☒	Eukaryotic cell lines
☒	☐	Palaeontology and archaeology
☐	☒	Animals and other organisms
☒	☐	Human research participants
☐	☒	Clinical data
☒	☐	Dual use research of concern

Methods

n/a	Involved in the study	
☒	☐	ChIP-seq
☒	☐	Flow cytometry
☒	☐	MRI-based neuroimaging

Antibodies

Antibodies used	α-SMA ?ab5694, abcam, USA, WB used at 1/400 dilution;ab124964, abcam, USA, IF used at 1/250 dilution, IHC used at 1/200 dilution?,<br>CCL5 (710001, invitrogen, USA;ab189841, abcam, USA, WB used at 1/1000 dilution, IHC used at 1/20 dilution),<br>phosphorylated (p)-NF-κB(Ser536) (ab28856, abcam, USA, IHC used at 1/100 dilution; #3033, CST, USA, WB used at 1/1000 dilution, IF used at 1/1600 dilution),<br>total NF-κB antibody (#8242, CST, USA, WB used at 1/1000),<br>RAD51 (ab133534, abcam, USA, IF used at 1/1000 dilution),<br>γ-H2AX (ab22551, abcam, USA, IF used at 1/200 dilution)
Validation	Validation data of the antibodies are available in supplier webpage.

Eukaryotic cell lines

Policy information about cell lines

Cell line source(s)	OV90, OVCAR3, A2780, OVCAR8, SKOV3, SW626 and HCC1937 were purchased from ATCC (Rockville, MD, USA), human lung fibroblast MRC-5 was obtained from the cell bank of the Chinese academy of sciences, Primary OC stromal fibroblasts were isolated and purified from fresh cancer tissues of OC patient. MRC5 was transformed into activated phenotype MRC5-CAF by TGF-β1 (50ng / ml).

Authentication	None of the cell lines used were authenticated.
Mycoplasma contamination	Mycoplasma testing (Lonza) is performed regularly in our institution, and all cell lines were tested once after thawing or isolation and before other experiments.
Commonly misidentified lines (See ICLAC register)	N/A

Animals and other organisms

Policy information about studies involving animals; ARRIVE guidelines recommended for reporting animal research

Laboratory animals	BALB/c nude mice
Wild animals	N/A
Field-collected samples	N/A
Ethics oversight	All animal experiments were conducted in compliance with approval of the Committee on the Ethics of Animal Experiments in Hubei province.

Note that full information on the approval of the study protocol must also be provided in the manuscript.

Clinical data

Policy information about clinical studies

All manuscripts should comply with the ICMJE guidelines for publication of clinical research and a completed CONSORT checklist must be included with all submissions.

Clinical trial registration	Not applicable.
Study protocol	Not applicable.
Data collection	Human biospecimens and related clinical data used in this article were acquired Tongji Hospital, Tongji Medical College, Huazhong University of Science & Technology during Jan. 2018 to Sep. 2019. All participants provided written informed consent at recruitment and the institutional ethics review committee of Tongji Hospital approved all study procedures for human subjects.
Outcomes	Not applicable.
